# A comparative study of carbonic anhydrase activity in lymphocytes from colorectal cancer tissues and adjacent healthy counterparts

**DOI:** 10.1080/14756366.2022.2085694

**Published:** 2022-06-13

**Authors:** Giulia Nannini, Viviana De Luca, Chiara D’Ambrosio, Andrea Scaloni, Antonio Taddei, Maria Novella Ringressi, Fabio Cianchi, Fabio Staderini, Clemente Capasso, Amedeo Amedei, Claudiu T. Supuran

**Affiliations:** a Department of Experimental and Clinical Medicine, University of Florence, Florence, Italy; b Institute of Biosciences and Bioresources, National Research Council, Napoli, Italy; c Proteomics, Metabolomics and Mass Spectrometry Laboratory, ISPAAM, National Research Council, Portici, Italy; d SOD of Interdisciplinary Internal Medicine, Azienda Ospedaliera Universitaria Careggi (AOUC), Florence, Italy; e Section of Pharmaceutical and Nutraceutical Sciences, Department of Neurofarba, University of Florence, Florence, Italy

**Keywords:** Carbonic anhydrase, isoforms, T cells, tumour-infiltrating lymphocyte, colorectal cancer

## Abstract

Several carbonic anhydrase (CA, EC 4.2.1.1) isoforms play an essential role in processes connected to tumorigenesis, as they efficiently accelerate the hydration of carbon dioxide to bicarbonate and proton. In this context, examples are CA IX and CA XII, which were proved to be upregulated in many solid malignancies. On the other hand, cancer and the immune system are inextricably linked, and targeting the immune checkpoints recently was shown to efficiently improve the treatment of malignancies. In this study, we have investigated the expression of CA isoforms in tumour-infiltrating lymphocytes (TILs) that, according to the immunosurveillance theory, were suggested to have a crucial role in the development of colorectal cancer (CRC). T lymphocytes isolated from healthy surrounding mucosa showed a higher CA activity compared to those present in tumour and peripheral blood in the same patients. CA I and II were confirmed as enzyme isoforms involved in the process, as determined by proteomic analysis of corresponding TIL samples. These preliminary findings suggest a dysregulation of the local immune response in the CRC tissues and a loss of effective anticancer mechanisms mediated by CAs therein.

## 1. Introduction

Colorectal cancer (CRC) is the world’s third most frequent malignancy and the second most deadly^1^. Twenty-five percent of newly diagnosed patients have metastatic disease, and 40% will develop metastases within a year^2^. Currently, the most popular conventional treatments for CRC include surgery, chemotherapy, and radiotherapy, which can be used in combination depending on the location and progression of cancer^3^. Targeting the immune system is one of the approaches that has revolutionised cancer treatment in the previous few decades^4^. In detail, immunotherapy tries to overcome the limitations of chemotherapy and radiotherapy by focussing on the immune system of the patient. Immune checkpoint inhibitors (ICIs) for programmed death receptor (PD-1) blockade have been approved for the CRC treatment with deficient mismatch repair (dMMR) or high microsatellite instability (MSI-H)^5^^,^^6^. For patients with proficient mismatch repair or microsatellite stability, however, immunotherapy using the anti-PD-1 monoclonal antibody did not have the expected impact. Despite the fact that ICIs have just recently been used in MSI-H/dMMR CRC patients, resistance to treatment has already been described^7^.

It is well known that solid tumours are characterised by the hypoxic microenvironment, extracellular acidosis, and chemoresistance. In nutrient-limited situations, tumour cells modify their metabolism by shifting the balance of energy production away from oxidative metabolism and towards a more glycolytic source^8^. The accumulation of lactic acid and carbon dioxide (CO_2_) caused by glycolytic metabolism contributes to a drop in extracellular pH. In order to survive, tumour cells must adapt to these settings, and several carbonic anhydrases (CAs, EC 4.2.1.1) play an essential role in this process^9^ since they accelerate the hydration of CO_2_ into bicarbonate (HCO_3_^–^) and proton (H^+^), a reaction essential to all living organisms^10–16^. Up to date, the CA superfamily is divided into eight CA classes, denoted by the Greek letters α, β, γ, δ, ζ, η, θ, and ι^10–14^^,^^17^. CA classes are spread out in a variegated way in plants, animals, bacteria, and archaea^10–14^. The genome of mammals, for example, only contains α-CAs, with 15 different isoforms (CA I-XV) that perform various tasks in different tissues and organs^10–14^.

Recent studies suggest that some CA isoforms, namely CA IX and CA XII, are upregulated in several solid malignancies^18^^,^^19^. For example, CA IX is found in only a few normal tissues, with almost complete exclusivity in the epithelium of the gastrointestinal tract^20^^,^^21^; on the other hand, this protein is expressed ectopically in a range of cancer tissues^18^^,^^22^, among which CRC^18^^,^^19^. By using CA II-deficient mice, a strong relationship between cellular control of acid-base balance and innate renal defence was discovered^23^. This finding may corroborate the existence of a possible link between CA II dysregulation and the immune system in cancer^24^. Despite this evidence, none investigated the expression of CAs in tumour-infiltrating lymphocytes (TILs) that, according to the immunosurveillance theory, were suggested to have a crucial role in developing tumours, as we have previously documented in human CRC^25^^,^^26^. In addition, growing evidence suggests that tumour mutation burden and TILs are linked to ICI response^27–29^. For these reasons, the aim of this preliminary study was to investigate the expression of CAs in TILs obtained from patients with CRC.

## 2. Materials and methods

### 2.1. Patients

The patients included in the study (P1-P18) and their clinico-pathological features are shown in Table 1.

**Table 1.** Clinical characteristics of CRC patients.

Patient ID	Sex	Age	Diagnosis	TNM	Stage
P1	M	71	Adenocarcinoma	–	–
P2	M	62	Adenocarcinoma	pT1a N0	I
P3	F	78	Adenocarcinoma	pT3 N0	IIa
P4	F	83	Adenocarcinoma	T3N0	2a
P5	M	78	Adenocarcinoma	pT3 N1b	IIIb
P6	M	58	Adenocarcinoma	pT3, pN1b	IIIb
P7	M	62	Adenocarcinoma	pT3 N0	IIa
P8	M	77	Adenocarcinoma	pT3 N0 M1	IVa
P9	F	53	Adenocarcinoma	pT1 N0	I
P10	M	85	Adenocarcinoma	pT3 N1b	IIIb
P11	F	85	Adenocarcinoma	T3N0	2a
P12	M	58	Adenocarcinoma	pT3, pN1b	IIIb
P13	M	84	Adenocarcinoma	pT2 N0	I
P14	M	74	Adenocarcinoma	pT3 N0	IIa
P15	F		Adenocarcinoma	–	–
P16	F	84	Adenocarcinoma	pT2 N1a	IIIa
P17	F	57	Adenocarcinoma	pT3 N0	IIa
P18	F	60	Adenocarcinoma	pT3, pN0	IIa

–, not detected.

### 2.2. Isolation of lymphocytes

Surgical specimens of CRC tissue were dissociated in order to isolate TILs. Tissue pieces from each patient were obtained from two different sites, namely central tumour (CT) and adjacent healthy mucosa (HM). Tissue samples were dissociated with the Tumour Dissociation Kit, human (Miltenyi Biotech, UK) in combination with the gentleMACS™ Octo Dissociator (Miltenyi Biotech, GmbH) to obtain a gentle and rapid generation of single-cell suspensions. In parallel, heparinised venous blood samples were collected and peripheral blood (PBMC) samples were isolated by density gradient centrifugation. Then, lymphocytes were magnetically isolated from dissociated CT, HM and PBMC samples with antihuman CD3 microbeads (Miltenyi Biotech, UK) using an AutoMACS Pro Separator device (Miltenyi Biotech, GmbH).

### 2.3. Protonography

An identical amount of lymphocyte proteins isolated from CT, HM, and PBMC samples from patients P1-P18 were mixed in a loading buffer for SDS-PAGE not containing 2-mercaptoethanol, and they were not boiled to avoid protein denaturation. Protein electrophoresis was performed as described by De Luca *et al.*^30^. After, the gel was subjected to protonography to detect the hydratase activity^30^. A parallel SDS-PAGE gel was run simultaneously in the same electrophoretic chamber. This gel was not used for investigating the hydratase activity, but for protein identification purposes; thus, it was stained with colloidal Coomassie blue.

### 2.4. Proteomic analysis

Gel slices associated with CA activity in the protonogram were manually excised from the colloidal Coomassie blue-stained gel, minced, and washed with water. Corresponding proteins were *in-gel* reduced, S-alkylated with iodoacetamide and digested with trypsin, as previously reported^31^. Individual protein digests were then analysed with a nanoLC-ESI-Q-Orbitrap-MS/MS platform consisting of an UltiMate 3000 HPLC RSLC nanosystem (Thermo Fisher Scientific, USA) coupled to a Q-ExactivePlus mass spectrometer through a Nanoflex ion source (Thermo Fisher Scientific)^32^. Peptides were loaded on an Acclaim PepMapTM RSLC C18 column (150 mm × 75 μm ID, 2 μm particles, 100 Å pore size; Thermo Fisher Scientific), and eluted with a gradient of solvent B (19.92/80/0.08 v/v/v water/acetonitrile/formic acid) in solvent A (99.9/0.1 v/v water/formic acid), at a flow rate of 300 nl/min. The gradient of solvent B started at 3%, increased to 40% over 40 min, raised to 80% over 5 min, remained at 80% for 4 min, and finally returned to 3% in 1 min, with a column equilibrating step of 30 min before the subsequent chromatographic run. The mass spectrometer operated in data-dependent mode using a full scan (*m/z* range 375–1500, a nominal resolution of 70,000, an automatic gain control target of 3,000,000, and a maximum ion target of 50 ms), followed by MS/MS scans of the 10 most abundant ions. MS/MS spectra were acquired in a scan *m/z* range 200–2000, using normalised collision energy of 32%, an automatic gain control target of 100,000, a maximum ion target of 100 ms, and a resolution of 17,500. A dynamic exclusion value of 30 s was also used. Duplicate analysis of each sample was performed to increase the number of identified peptides/protein coverage.

MS and MS/MS raw data files per sample were merged for protein identification into Proteome Discoverer v. 2.4 software (Thermo Scientific), enabling the database search by Mascot algorithm v. 2.6.1 (Matrix Science, UK) with the following parameters: UniProtKB human protein database (11/2020, 214889 sequences) including the most common protein contaminants; carbamidomethylation of Cys as fixed modification; oxidation of Met, deamidation of Asn and Gln, and pyroglutamate formation of Gln as variable modifications. Peptide mass tolerance and fragment mass tolerance were set to ± 10 ppm and ± 0.05 Da, respectively. Proteolytic enzyme and maximum number of missed cleavages were set to trypsin and 2, respectively. Protein candidates assigned on the basis of at least one sequenced peptides with Mascot score ≥30 were considered confidently identified. Definitive peptide assignment was always associated with manual spectra visualisation and verification.

## 3. Results and discussion

### 3.1. CA activity and isoform detection in lymphocytes

#### 3.1.1. CA activity

A cohort of 18 patients (10 males and 8 females) with intestinal adenocarcinoma was included in this study (Table 1).

Colorectal tumour tissues and healthy mucosal tissues were collected during surgery and analysed by pathologists to determine TNM (tumour, nodes, and metastases) stage. In parallel, heparinised venous blood samples were collected from patients and peripheral blood (PBMC) samples were isolated. Then, lymphocytes were isolated from the above-mentioned CT and HM tissues as well as PBMC samples to investigate corresponding hydratase activity and identify CA isoforms possibly responsible for it.

First, the CO_2_ hydratase activity in lymphocytes was investigated with protonography, a technique developed by our group^30^. It allows the measurement of pH change in the gel (protonogram) caused by the catalytic conversion of CO_2_ into HCO_3_^–^ and H^+^. The pH indicator bromothymol blue was used to stain the protonogram. This dye appears blue when deprotonated but becomes yellow when protonated. The generation of hydrogen ions by the enzyme hydratase activity lowers the pH value of the solution until the dye colour transition point is reached (pH 6.8).

**Figure 1.**
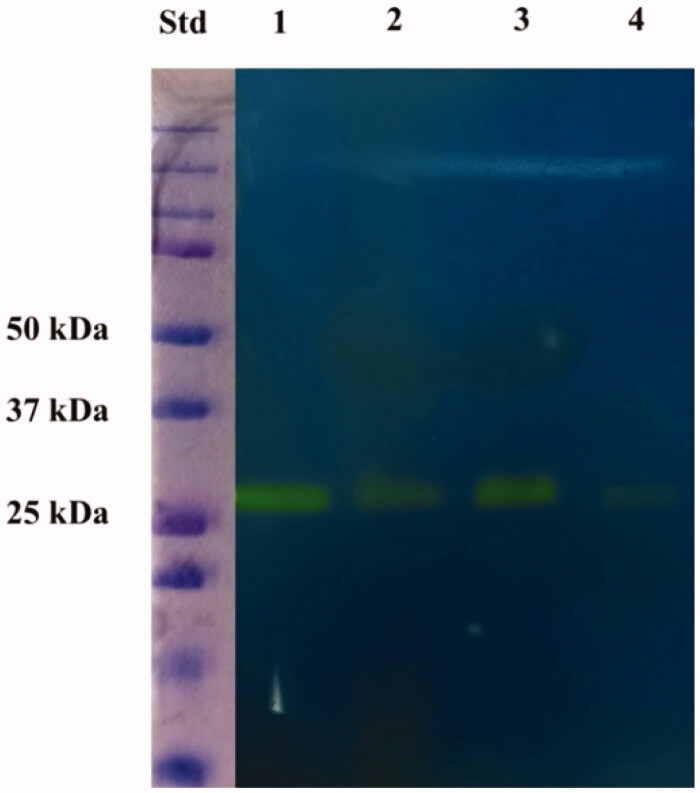
Protonography of whole lymphocyte extracts prepared from tumour and adjacent healthy tissues as well as from peripheral blood of a patient with intestinal adenocarcinoma included in this study. The gel was run under denaturing but non-reducing conditions. Protonography showed a yellow band migrating with a rough molecular mass of about 26 kDa, which corresponds to the hydratase activity. Legend: Lane Std, molecular markers (from bottom to the top: 25 kDa, 37 kDa, 50 kDa, and others); lane 1, commercial bovine CA used as positive control; lane 2, lymphocyte collected from the tumour tissue; lane 3, lymphocyte collected from adjacent healthy tissues; lane 4, lymphocyte collected by PBMCs.

Figure 1 shows an exemplificative protonogram obtained with lymphocyte samples collected from CT and HM as well as PBMCs of patient P1; identical amounts of lymphocytes were analysed in each protonogram. Very similar results were obtained in the case of the other patients, which always showed a similar staining pattern (data not shown). The gel portion of the gel coloured in yellow corresponded to the region where the protein(s) responsible for the hydratase activity migrated during electrophoresis. A parallel gel running different amounts of commercial bovine CA was used to successfully evaluate the linearity of the staining intensity with respect to the loaded protein.

Lymphocyte protonograms were analysed using image processing technologies to evaluate the relative representation of the hydratase activity in the different samples. In the case of patient P1, densitometric quantification revealed that lymphocytes of the vicinal healthy tissues had a hydratase activity that was about 3.1- and 17.1-fold higher than that of CT and PBMC counterparts, respectively. Accordingly, the hydratase activity of the lymphocytes coming from CT was about 5.5 times higher than that of PBMC. Very similar results were obtained in the case of the other patients, thus demonstrating that lymphocytes of HM samples had an overall average hydratase activity that was about 3.0 ± 0.5 and 17.0 ± 1.1 fold higher than that of CT and PBMC counterparts, respectively. Based on these results, we hypothesised that the fine-tuning of CO_2_, HCO_3_^–^, and H^+^ in the lymphocytes from tumoral tissues and PBMCs might be affected with respect to counterparts from healthy tissues. This observation was in good agreement with previous literature data reporting that systemic acidosis may impair immune function. Thus, cancer-associated inflammation may contribute to genomic instability, epigenetic change, cancer cell proliferation, an increase in antiapoptotic pathways, angiogenesis, and ultimately cancer propagation^23^^,^^24^.

#### 3.1.2. Proteomic analysis of gel portions associated with CA activity

In order to identify the protein(s) present in the electrophoretic bands associated with the hydratase activity measured in the protonogram of lymphocytes from TC and vicinal HM as well as from PBMCs, corresponding gel portions were cut from the gels and subjected to proteomic analysis, as described in the experimental section. Experiments were performed in parallel on bands showing positive staining in the protonogram of lymphocytes from patient P1. In both cases, several protein components were identified in the different samples, among which some carbonic anhydrase isoforms, here considered responsible for the observed enzymatic activity based on corresponding biochemical characteristics. Supplementary Table S1 summarises the proteomic results for all analysed samples, while Table 2 shows the carbonic anhydrase isoforms identified therein.

**Table 2.** Protein identification details of gel portions associated with CO_2_ hydratase activity through protonography.

Sample	Accession	Gene name	Description	Sequence coverage (%)	Peptides (number)	PSMs	Unique Peptides	Amino acids	Score (Mascot)
Patient P1-HM	E5RHP7	CA1	Human carbonic anhydrase I	22	4	10	4	251	382
	P22748	CA4	Human carbonic anhydrase IV	9	2	4	2	312	134
	V9HW21; P00918	CA2	Human carbonic anhydrase II	32	6	18	6	260	502
Patient P1-CT	V9HW21; P00918	CA2	Human carbonic anhydrase II	3	1	1	1	260	45
Patient P1-PMBCs	E5RHP7	CA1	Human carbonic anhydrase I	39	7	18	7	251	627
	V9HW21; P00918	CA2	Human carbonic anhydrase II	25	5	9	5	260	379

It was evident that CA I and II were the isoforms associated with the ascertained enzymatic activity in all cases. This result was not surprising since these proteins were already identified as CA isoforms present in human T and NK cells^33–36^. Furthermore, proteomic identification parameters of these proteins in tumour and vicinal healthy tissue as well as in PBMC samples well paralleled enzymatic activities detected during protonographic measurements, further confirming that a protein representation decrease of these components was associated with lymphocyte degeneration characteristics. Together with isoforms CA IV, VII, XII, and XIII, CA I and CA II were already identified as highly represented in the colon and rectum of human patients^33–36^.

## 4. Conclusions

Carbonic anhydrases were proven to act as significant pH mediators in tumour cells by regulating HCO_3_^–^ and H^+^ concentrations. Among the 15 CA isoforms present in humans, two CA isozymes have attracted significant attention as anticancer targets, namely CA IX and CA XII^37^. These two transmembrane proteins are significantly expressed in solid tumours and, through their catalytic extracellular domain, were proved to modulate the pH value of the tumour microenvironment, increasing cancer cell survival and proliferation^37^. On the other side, few data are reported on the role of other CA isoforms in tumours^38^. For example, CA I and CA II were shown to be upregulated in some cancer types, such as prostate, breast, melanomas, bladder, thyroid, breast, lung, liver, gliomas, renal cell carcinomas, and head and neck. Conversely, pancreatic, colorectal, gastric, and gastrointestinal stromal cancerous tissues showed a downregulation of the CA II protein expression, which was associated with cancer aggressiveness^38^. In addition, CA XIII downregulation was found in CRC samples, even though the clinical significance of these findings has not been investigated.

In this context, the combined use of protonography and proteomic procedures has allowed us originally to evaluate the representation of CA activity and isoforms in TILs, which play a critical role in the development of malignancies such as CRC. As a result, we have here demonstrated that T lymphocytes isolated from the healthy surrounding mucosa had a higher hydratase activity than those present in tumour and peripheral blood. Notably, we also have documented that CA I and II were the isoforms responsible for the different enzymatic activity we measured in TC, HM, and PBMC samples of the enrolled patients.

Previous studies have detailed that CAs are variably represented in different immune cell types, such as B cells, NK, monocytes, and T cells. Noteworthy, CA I and CA II expression was observed in both CD4^+^ and CD8^+^ activated T cells^39^. In addition, several T cell subsets, such as Treg, Th1, Th17, and Th2 cells, which we have previously demonstrated to have a crucial role in the development of CRC and other gastrointestinal cancers, were proved to express CA I and CA II^26^^,^^40^^,^^41^. Accordingly, we can realistically hypothesise that the downregulation CAs can affect systemic acidosis, thus impairing the immune function and the related cancer-associated inflammation, promoting genomic instability, epigenetic change, cancer cell proliferation, angiogenesis, and ultimately cancer spread^23^^,^^24^. Interestingly, lymphocytes were shown to promote a CA-dependent epithelial HCO_3_^–^ secretion as a critical host defence mechanism in other immunity-related contexts, such as during human prostatitis and lung bacterial infection^42^^,^^43^. Based on the data reported in this study, we could suppose that the immune system is damaged at the tumour site and cannot promote HCO_3_^–^ secretion, thus leading to a drastic reduction of HCO_3_^–^ levels compared to the corresponding adjacent healthy tissue. The low hydratase activity of CA I and CA II and a corresponding reduction of HCO_3_^–^ secretion in CRC-infiltrating T lymphocytes may suggest a dysregulation of the local immune response and a consequent loss of effective anticancer mechanisms. Further studies are necessary to corroborate this preliminary observation and elucidate the ongoing processes linking acidosis and CRC development.

## Supplementary Material

Supplemental Material
